# Cor Triatriatum Sinistrum

**DOI:** 10.5935/abc.20170138

**Published:** 2018-01

**Authors:** Hitesh Raheja, Vinod Namana, Norbert Moskovits, Gerald Hollander, Jacob Shani

**Affiliations:** Maimonides Medical Center, NY - USA

**Keywords:** Cor Triatriatum, Heart Defects, Congenital, Echocardiography, Transesophageal

**Figure 1:**
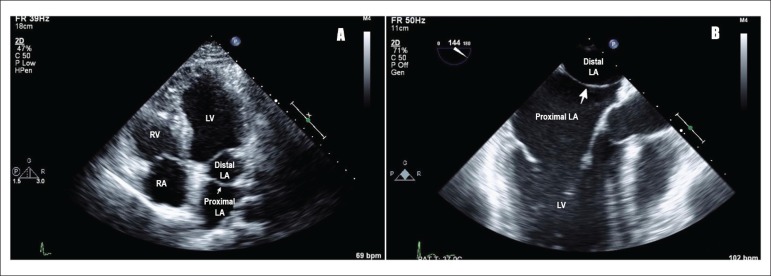
A) Transthoracic echocardiogram showing cor triatriatum: proximal and distal left atrium separated by a membrane (Pointing white arrow), LA: left atrium; LV: left ventricle; RV: right ventricle; RA: right atrium. B) Transesophageal echocardiogram showing cor triatriatum: proximal and distal left atrium separated by a membrane (Pointing white arrow), LA: left atrium; LV: left ventricle.

A 25-year-old male presented to clinic with complaints of palpitations. Transthoracic echocardiogram (TTE) showed presence of a membrane in left atrium suggestive of cor triatriatum [Figure 1A]. This finding was confirmed with transesophageal echocardiogram (TEE), which revealed a membrane in the left atrium attaching at the Coumadin ridge and the atrial septum, just caudal to the fossa ovalis [Figure 1B].

Cor triatriatum sinistrum (CTS) is a rare congenital malformation in which the left atrium is divided into two chambers by a fenestrated fibro-muscular septum. The posterior proximal left atrial chamber receives the pulmonary veins and the anterior distal left atrial chamber contains the mitral valve and left atrial appendage. Cor triatriatum accounts for 0.1% to 0.4% of congenital heart defects. This defect generally manifests during infancy and early childhood. However, some cases present well into adulthood as in our patient. The most common presenting symptoms are dyspnea, orthopnea, hemoptysis, palpitations and chest pain. Although cor triatriatum can be an isolated lesion as in our patient, it is frequently associated with other congenital cardiovascular anomalies, most often ASD. Echocardiography is the mainstay for diagnosis. CTS is first suspected by the presence of a linear structure in the left atrium on TTE. TEE is used for better visualization of the membrane, measurement of gradients across the membrane and to recognize ASD. In symptomatic patients, management consists of resection of the diaphragm and correction of the associated congenital heart defects. Conservative approach is often implemented in asymptomatic adults.

